# Masking effects on subjective annoyance to aircraft flyover noise: An fMRI study

**DOI:** 10.1002/hbm.25016

**Published:** 2020-05-07

**Authors:** Nishuai Yu, Jun Cai, Xuanyue Xu, Yining Yang, Junfeng Sun

**Affiliations:** ^1^ School of Environmental Science and Engineering, Shanghai Jiao Tong University Shanghai China; ^2^ Shanghai Med‐X Engineering Research Center, School of Biomedical Engineering, Shanghai Jiao Tong University Shanghai China; ^3^ Brain Science and Technology Research Center Shanghai Jiao Tong University Shanghai China

**Keywords:** anterior cingulate cortex (ACC), fMRI, functional connectivity, noise annoyance, prefrontal cortex (PFC), sound masking

## Abstract

Sound masking, a new noise control technology, has been applied to improve subjective perception of noise in recent years. However, the neural mechanisms underlying this technology are still unclear. In this study, 18 healthy subjects were recurited to take subjective annoyance assessments and fMRI scanning with the aircraft noise and the masked aircraft noise. The results showed that the noise annoyance was associated with deficient functional connectivity between anterior cingulate cortex (ACC) and prefrontal cortex and exceeded brain activation in ACC, which might be explained as compensation. The sound masking led to significantly strong activation in the left medial frontal cortex and right medial orbital frontal cortex, which were associated with happy emotion induced by sound masking. This study offered new insights on the underlying neural mechanisms of sound masking effects.

## 1 INTRODUCTION

Noise pollution is one of the most harmful environmental issues. Chronic exposure in noise situation can cause annoyance and the related harms (Basner et al., 2014), such as hearing loss (Lie et al., 2016), hypertension (Dimakopoulou et al., 2017; A. S. Evrard, Lefevre, Champelovier, Lambert, & Laumon, 2017), stroke (Floud et al., 2013), and cardiovascular disease (Correia, Peters, Levy, Melly, & Dominici, 2013; A.‐S. Evrard, Bouaoun, Champelovier, Lambert, & Laumon, 2015). To reduce the noise impacts, noise control technologies were developed in the past decades (Audi, 1992; Defrance & Jean, 2003; Menounou & You, 2004). However, these traditional noise control technologies were little effective to some noise with high energy and broadband spectra, such as aircraft noise, industrial noise, and traffic noise. Although less noisy aircrafts and engines have been designed, aircraft noise annoyance is still a long‐standing problem not only for the exposed people nearby (Babisch et al., 2009; Schreckenberg, Meis, Kahl, Peschel, & Eikmann, 2010), but also for the traditional treatment methods (Kuznetsov, 2003; Rodriguez‐Diaz, Adenso‐Diaz, & Gonzalez‐Torre, 2017). So a trend of aircraft noise control is to improve the perception instead of the noise energy attenuation (Hatfield et al., 2002). Sound masking is such a technology whose essence is to improve the subjective feelings to noise by introduction of the masking sound. As the supplement of traditional methods, the sound masking was recently adopted in sound quality treatments (Bolin, Nilsson, & Khan, 2010; Cai, Liu, Yu, & Liu, 2019; Nilsson, Alvarsson, Radsten‐Ekman, & Bolin, 2010; Radsten‐Ekman, Axelsson, & Nilsson, 2013). For example, the natural water sounds could be utilized to mask road‐traffic noise and improve the soundscape quality (Axelsson, Nilsson, Hellstrom, & Lunden, 2014). Sound masking could also be used to reduce discomfort feelings of dental treatment sounds (Suhara, Ikefuji, Nakayama, & Nishiura, 2013). However, these studies were mainly limited to the domains of acoustics and psychoacoustics. The fundamental neural mechanisms underlying sound masking are still not well understood.

In recent years, functional magnetic resonance imaging (fMRI) has been widely developed and used to investigate the brain response of auditory sensation. Some research focused on the pathology neurological studies, such as the difference of brain response to auditory stimuli between patients and healthy people (Behroozmand et al., 2018). These research figured out that the neural mechanisms of auditory related diseases like tinnitus (Hullfish, Abenes, Yoo, De Ridder, & Vanneste, 2019), schizophrenia (Schirmer et al., 2009), and panic disorder (Schwarzmeier et al., 2019). Others focused on the auditory perception and analyzed the correlations between brain response and some fundamental acoustic indexes, such as sound pressure level (SPL; Jancke, Shah, Posse, Grosse‐Ryuken, & Muller‐Gartner, 1998), frequency (Wilson, Melcher, Micheyl, Gutschalk, & Oxenham, 2007) and timbre (Deike, Gaschler‐Markefski, Brechmann, & Scheich, 2004). Moreover, fMRI technology was also used in the research on the neural mechanisms of musical tone identification ability (McKetton, DeSimone, & Schneider, 2019), sound source localization (Trapeau & Schoenwiesner, 2018), and the individual differences in music reward sensitivity (Martinez‐Molina, Mas‐Herrero, Rodriguez‐Fornells, Zatorre, & Marco‐Pallares, 2019). However, there has been no research on the brain response and neural mechanisms of sound masking by fMRI technology.

The aim of this study is to explore how the sound masking improves the subjective feelings from the perspective of brain response, and to analyze the psycho‐physiological relationship and neural mechanisms of sound masking by fMRI technology. The music clip, violin concerto “Butterfly Lovers,” was selected to mask the aircraft flyover noise in this study. Eighteen subjects were recruited to carry out both fMRI scanning and subjective annoyance assessments with aircraft noise and the masked aircraft noise. The contrasts in the brain activations and the seed‐maps of the target seed regions under two stimuli were analyzed.

## 2 MATERIALS AND METHODS

### 2.1 Subjects

Eighteen right‐handed young adults (nine males, nine females; mean age = 26 years, standard deviation of age = 8.08 years) without neurological diseases or hearing impairment were enrolled to participate this study. Each subject was carried out an fMRI scanning and a subjective annoyance assessment (Fields et al., 2001), respectively. The fMRI scanning was approved by the ethics committee of Shanghai Mental Health Center. The written informed consents were obtained from all subjects.

### 2.2 Auditory stimuli

**FIGURE 1:**
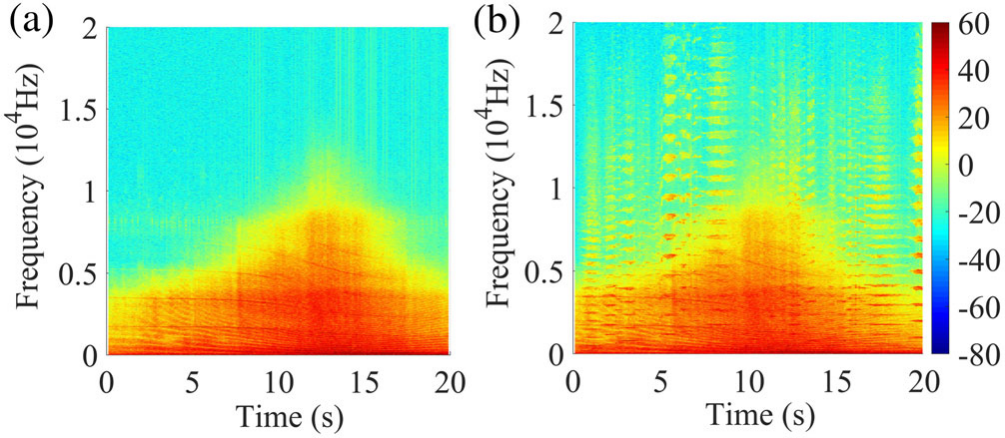
(a) The spectrogram of the aircraft flyover noise; (b) The spectrogram of the masked aircraft noise. The color bar indicates the magnitude of sound energy

The aircraft flyover noise (Figure 1a) signal was recorded at the neighborhood nearby Hongqiao Airport (Shanghai, China) by binaural recorder (BR2022) with 44.1 kHz sampling rate and 24‐bit resolution. The A‐weighted equivalent sound pressure Level (L_Aeq_) of aircraft flyover noise signal, measured by B&K 2270 analyzer on site, was around 80 dBA. To mimic the real situation, the SPL of the aircraft noise signal were set to 80 dBA (L_Aeq_, 20s; Table 1). In our own preliminary subjective annoyance evaluation experiment, a piece of music clipped from the Chinese violin concerto “Butterfly lovers” music gained the most effective masking effect on aircraft noise among the cases of other masking sounds (i.e., fountain, waterfall, wind, music clipped from Romance De Amor; unpublished results) and thus was selected as the masking sound in this study. The prior study showed that there was the detection threshold difference when the pure signal was masked by a narrow‐band noise with different conditions (Wack, Polak, Furuyama, & Burkard, 2014). This reminded us the importance of SPL difference between the noise and masking sound. According to the previous study (Jeon, Lee, You, & Kang, 2010; Shu, Song, & Zhou, 2018), the masking sound with similar SPL to or not less than 3 dB below the SPL of the noises would gain the most effective masking effect. What's more, in our own preliminary subjective annoyance evaluation experiment, “Butterfly lovers” music of 0 dBA SPL difference from the aircraft noise (SPL 80 dBA) gained the best masking effect among ones of −3， 0, and 3 dBA SPL difference (unpublished results). Therefore, the aircraft noise (80 dBA) and one combined with “Butterfly lovers” music of 80 dBA by Adobe Audition CC 2017 were used as the auditory stimuli in the following study (Table 1). The spectrogram of the aircraft flyover noise and the masked aircraft noise showed that the frequency range of the music covered that of the aircraft flyover noise (Figure 1), which was necessary for effective sound masking.

**TABLE 1:** The parameters of two auditory stimuli

Stimuli	Duration time (s)	Experiment signal	L_Aeq_(dBA)
1	20	Aircraft noise	80
2	20	Masked aircraft noise	83

### 2.3 Subjective annoyance evaluation

The subjective annoyance evaluation experiment was conducted in a quiet audiometric room. To avoid the simplity, these two stimuli were contained in 10 other signals (five sounds such as fountain, waterfall, wind, birdcall, music clipped from Romance De Amor, and these five sound masked aircraft noise, respectively). All the sounds were presented through Otometrics ER‐2 in‐ear earphone in a random sequence. According to the standard of noise annoyance survey formulated by the International Commission on the Biological Effects of Noise (ICBEN; Fields et al., 2001), a 5‐scale and 10‐point verbal scale question was presented on the scoring window. The five scales (from 0 to 10) denote “Not at all,” “Slightly annoyed,” “Moderately annoyed,” “Very annoyed,” and “Extremely annoyed,” respectively (Preis, Kaczmarek, Wojciechowska, Zera, & Fields, 2003). The subjects were required to mark the annoyance values ranged from 0 to 10 based on their annoyance perception. The annoyance rating data were collected by the computer and further analyzed.

### 2.4 fMRI data acquisition

A block design paradigm was adopted in this study. Two sessions were established for Stimulus 1 and Stimulus 2, respectively. In either session, the stimuli were repeated seven times with 20s' interval. There was a 1‐min break between the two sessions. The stimulus was presented to the participants through Sensimetrics S14 insert earphone (Huth, de Heer, Griffiths, Theunissen, & Gallant, 2016) inside the soundproof earmuff. This promised high‐quality stimuli presentation and avoided the noise impact from the MRI scanner. The blinder patch was used to isolate visual stimuli which were irrelevant to the experiment. All the subjects were required to keep awake and concentrate on the stimuli.

The fMRI data were collected using a 3.0‐Tesla system (Magnetom Verio, Siemens, Munich, Germany) with a 16‐channel head coil. A soft foam padding was plugged tightly to decrease head motion. Anatomical scans were acquired using a T1‐weighted multi‐echo MPRAGE sequence with the following parameters: echo time (TE) = 3.5 ms; repetition time (TR) = 2,300 ms; flip angle (FA) = 9°; field of view (FOV) = 256 mm*256 mm; slice thickness = 1 mm; voxel size = 1*1*1mm^3^. Functional scans were acquired using a gradient echo sequence with the following parameters: echo time (TE) = 30 ms; repetition time (TR) = 2000 ms; flip angle (FA) = 90°; field of view (FOV) = 220 mm*220 mm; slice thickness = 4 mm; voxel size = 3.4*3.4*4mm^3^.

### 2.5 fMRI data preprocessing

The data were preprocessed by the software Dpabi (Yan, Wang, Zuo, & Zang, 2016). Volumes were corrected for time delay and realigned to the first volume. Head motion parameters for each volume were calculated by estimating the angular rotation on each axis and the movements in each direction. The maximum displacement was set to a 3 mm movement in any direction (*x*, *y*, *z*) and a 3° spin on each axis (*x*, *y*, *z*). Using the parameters estimated during linear coregistration, the motion‐corrected functional volumes were normalized to the individuals' structural images. All the images were resampled into 3 × 3 × 3 mm^3^ voxels. Finally, all the data were smoothed with a Gaussian kernel of 6 × 6 × 6 mm^3^ FWHM.

### 2.6 Statistical analysis of activation area

First, a one‐sample *t*‐test was carried out for each session using SPM12 (https://www.fil.ion.ucl.ac.uk/spm/). The subjects' brain activation areas and activation level were acquired. Second, a paired *t*‐test was applied between the two sessions to analyze the main differences of subjects' brain acitvation induced by the auditory stimuli with and without masking. Brain areas with obvious changes in brain activation level were figured out and their peak voxels were further analyzed. For each subject, the *T*‐values (the statistical measure of the signal that indicates brain activation level) of these peak voxels were extracted. A correlation analysis was finally conducted between the *T*‐values and subjective annoyance scoring. The anatomical automatic labeling (AAL) areas (Collins et al., 1998) and the Brodmann areas (Brodmann, 1909) were applied to present these brain areas.

Furthermore, functional connectivity analysis was performed. First, the linear trend was removed. The peak voxels in the activation areas acquired in the previous activation analysis were selected as the seed points. A ball with 6 mm redius around the seed point was defined as a seed region. For each subject, the mean fMRI time series of each seed region was extracted and correlated with the fMRI time series of every voxel in the whole brain. Fisher's *r*‐to‐*z* transform was applied for the normality of the correlation coefficients. The functional connectivity map of each seed region was created through a one sample *t*‐test (false discovery rate [FDR] corrected, *q*‐value = 0.001). A paired t‐test was applied between the two maps to analyze the main differences of subjects' functional connectivity induced by the auditory stimuli with and without masking.

## 3 RESULTS

### 3.1 Subjective annoyance scoring

**FIGURE 2:**
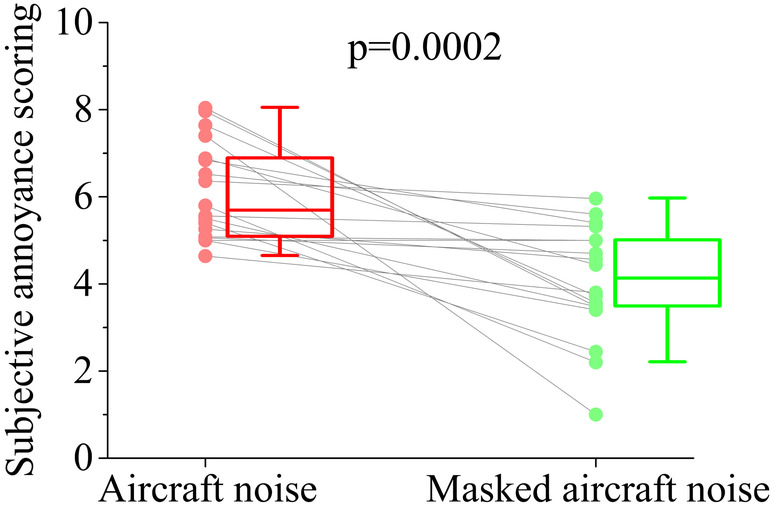
The subjects' annoyance scoring for the aircraft noise and the masked aircraft noise. Each dot represents the annoyance scoring of the two stimuli marked by the subjects

The subjects' annoyance scoring for the aircraft noise and the masked aircraft noise were shown in Figure 2. Paired *t*‐test analysis revealed that subjects' annoyance scoring for the aircraft noise was significantly higher than that for the masked aircraft noise (*p*‐value = .0002). This suggested that the masking sound we chose was effective to the aircraft noise. The masked aircraft noise was qualified as the stimulus in our study.

### 3.2 Brain activation

**FIGURE 3:**
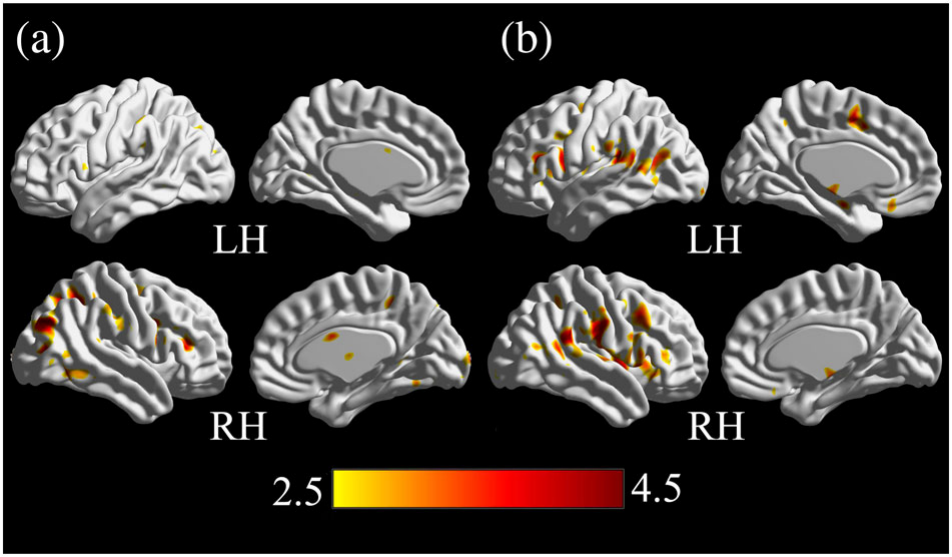
(a) Subjects' brain activation areas for the aircraft noise; (b) Subjects' brain activation areas for the masked aircraft noise

Brain activation areas were detected by comparing the fMRI data at resting state and under the two stimuli. The upper limit of the threshold was set to 0.001 (*p* < .001) and the lower limit of the cluster size was set to 10 (cluster size >10) in the analysis. For the aircraft noise, large clusters (cluster size ≥20) of activation appeared in the right medial occipital gyrus and right ACC. Small clusters (cluster size <20) of activation were observed in the right frontal lobe and right temporal lobe. For the masked aircraft noise, large clusters of activation appeared in the right postcentral gyrus and left superior temporal gyrus (STG). Small clusters of activation appeared in the bilateral inferior frontal cortex (Figure 3).

**FIGURE 4:**
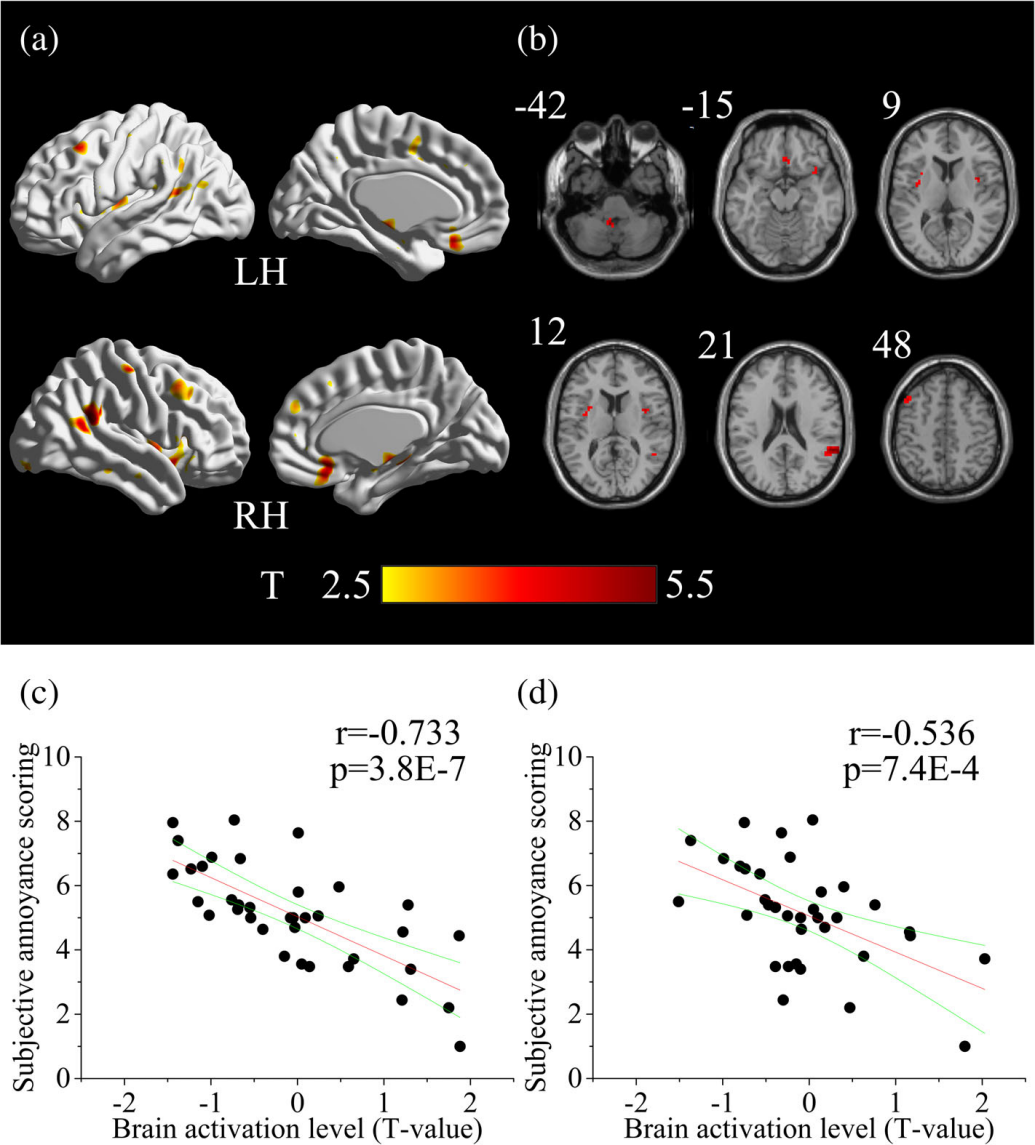
(a,b) The cortical surface and the slice view of the brain areas with stronger activation in the case of masked aircraft noise compared with the aircraft noise. In panel (b), the number at the left corner in the slice view stands for the *z*‐coordinate of the brain slice. (c,d) Correlation between the brain activation level and subjective annoyance scoring. In panel (c), correlation of left medial frontal cortex (*x* = −45, *y* = 21, *z* = 48), *r* = −.733; in panel (d), correlation of right orbital medial frontal cortex (*x* = 3, *y* = 27, *z* = −15), *r* = −.536

Refer to the brain activation under the aircraft noise, the paired *t*‐test revealed that the brain activation in the right temporal gyrus, left medial frontal cortex, right medial OFC and bilateral insula were significantly stronger (peak *T*‐value >2.5) under the masked aircraft noise (Table 2, Figure 4a,b). Correlation analysis between subjects' *T*‐values and subjective annoyance scoring was further performed. There was a significant negative correlation between the *T*‐values of two frontal cortical regions and subjective annoyance scorings (Figure 4c,d).

**TABLE 2:** Brain areas with stronger activation under the masked aircraft noise compared with the aircraft noise

AAL areas	Brodmann areas	Peak *T*‐value	*x*	*y*	*z*	Voxels
Temporal_sup_R	22	5.562	66	−45	21	69
Temporal_Mid_R	21	3.151	51	−54	18	
Insula_R	13	3.407	39	3	9	20
		3.222	36	12	−12	
Frontal_Med_Orb_R	11	3.167	3	27	−15	21
Frontal_Med_2_L	9	3.147	−45	21	48	11
Insula_L	13	2.955	−30	6	12	16
		2.938	−36	−6	9	
Cerebelum_9_L		3.802	−6	−48	−42	16

**FIGURE 5:**
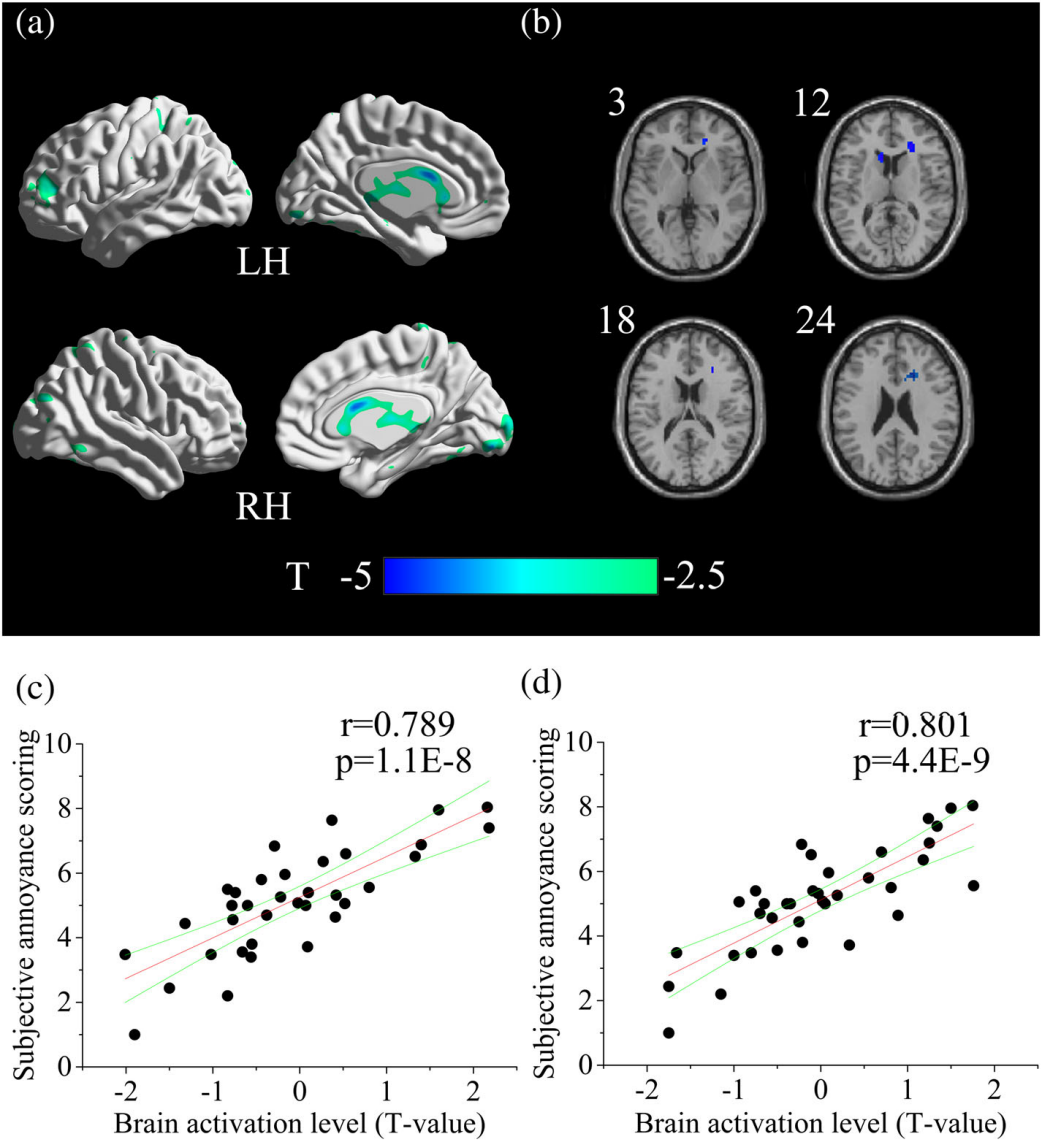
(a,b) The cortical surface and the slice view of the brain areas with weaker activation in the case of masked aircraft noise compared with the aircraft noise. In panel (b), the number at the left corner in the slice view stands for the z‐coordinate of the brain slice. (c,d) Correlation between the brain activation level and subjective annoyance scoring. In panel (c), correlation of left ACC (*x* = −9, *y* = 21, *z* = 12), *r* = .789; in panel (d), correlation of right ACC (*x* = 21, *y* = 21, *z* = 24), r = .801

On the contray, refer to the brain activation under the aircraft noise, the paired *t*‐test revealed that the brain activation in the bilateral ACC, right caudate and right medial frontal cortex were significantly weaker (peak *T*‐value <−2.5) under the masked aircraft noise (Table 3, Figure 5a,b). Furthermore, the *T*‐values of the bilateral ACC had a significant positive correlation with subjective annoyance scoring (Figure 5c,d).

**TABLE 3:** Brain areas with weaker activation under the masked aircraft noise compared with the aircraft noise

AAL areas	Brodmann areas	Peak *T*‐value	*x*	*y*	*z*	Voxels
Cingulate_Ant_R	24	−4.961	21	21	24	25
Cingulate_Ant_L	24	−4.829	−9	21	12	12
Caudate_R		−4.791	24	33	12	13
Frontal_Mid_2_R		−4.727	27	27	18	11
Cingulate_Ant_R		−4.293	21	39	3	15

### 3.3 Functional connectivity

Acoording to the above correlation analysises, the brain activitation level of four brain areas such as left medial frontal cortex, right orbital medial frontal cortex, and bilateral ACC, had significant correlations with subjective annoyance scoring. To figure out their related neural network, their peak voxels were extracted and used as the seed points to generate seed‐maps respectively. Furthermore, the functional connectivity between ACC and PFC was found to have a close relationship with negative emotions (Carballedo et al., 2011; Szekely, Silton, Heller, Miller, & Mohanty, 2017). The neural network including OFC, inferior occipital gyrus (IOG), parahippocampal gyrus (PHG), and bilateral STG was associated with happiness caused by music (Bogert et al., 2016). The functional connnectivity above was the object of our study.

**FIGURE 6:**
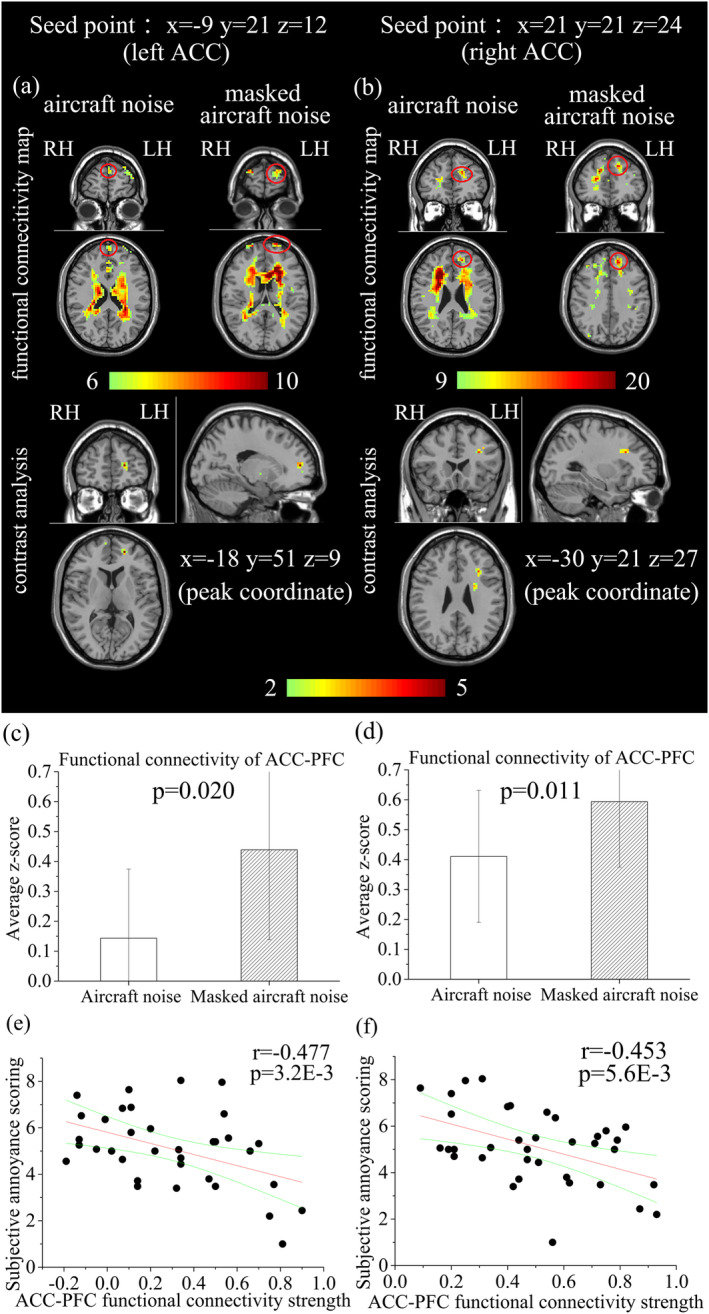
(a,b) The functional connectivity maps (*p* < .001, cluster size>10) of the seed regions in the bilateral ACC (PFC was circled in the functional maps) and the peak coordinates of PFC in the contrast analysis. (c) The strength of functional connectivity between the left ACC seed (*x* = −9, *y* = 21, *z* = 12) and the left PFC (x=‐18, y=51, z=9) across subjects. (d) The strength of functional connectivity between the right ACC seed (*x* = 21, *y* = 21, *z* = 24) and the left PFC (x=‐30, y=21, z=27) across subjects. (e) Correlation between the strength of ACC‐PFC functional connectivity and subjective annoyance scoring for the left ACC seed (*x* = −9, *y* = 21, *z* = 12). (f) Correlation between the strength of ACC‐PFC functional connectivity and subjective annoyance scoring for the right ACC seed (*x* = 21, *y* = 21, Z = 24). In panels (e) and (f), each dot represents one subject for aircraft noise or masked aircraft noise

**FIGURE 7:**
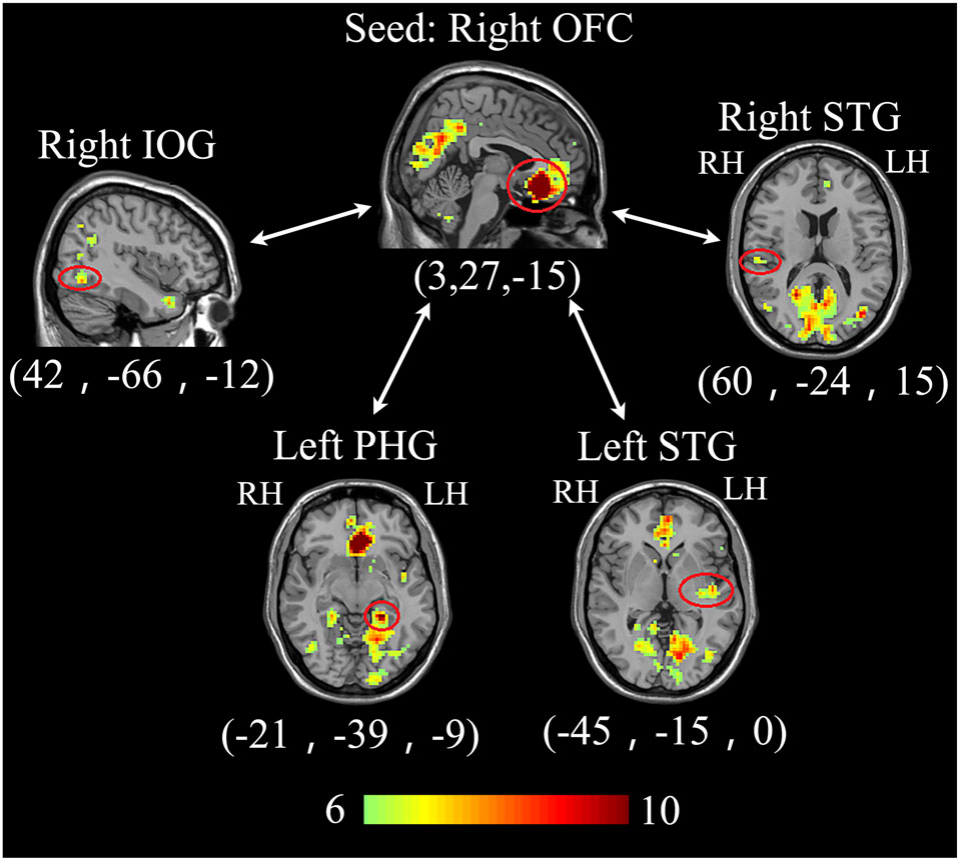
The neural network (*p* < .001,cluster size >10) of the seed region in the right OFC. IOG, inferior occipital gyrus; PHG, parahippocampal gyrus; STG, superior temporal gyrus

In the seed‐maps of bilateral ACC, the ACC‐PFC functional connectivity was observed under both aircraft noise and masked aircraft noise (Figure 6a,b). It was stronger under the masked aircraft noise than under the aircraft noise (Figure 6c,d). Futhermore, the strength of ACC‐PFC functional connectivity had a significant negative correlation with the subjective annoyance scoring (Figure 6e,f). In the seed‐maps of the two frontal cortical regions, the OFC had a significant functional connectivity with IOG, PHG, and bilateral STG (Figure 7) under the masked aircraft noise.

## 4 DISCUSSION

In the present study, the contrast analysis showed different brain activation under the aircraft noise and masked aircraft noise. In particular, the right STG, bilateral insula, left medial frontal cortex and right medial OFC showed a stronger activation under the masked aircraft noise than under the aircraft noise (Table 2, Figure 4a,b). The activation level in left medial frontal cortex and right medial OFC presented a significant negative correlation with the subjective annoyance scoring (Figure 4c,d). Caudate nucleus (CN) and bilateral ACC showed a weaker activation under the masked aircraft noise than under the aircraft noise (Table 3, Figure 5a,b). The activation level in bilateral ACC presented a significant positive correlation with the subjective annoyance scoring (Figure 5c,d). In the functional connectivity analysis, the strength of ACC‐PFC functional connectivity were negatively associated with subjective annoyance scoring (Figure 6e,f). The OFC was included in a network with IOG, PHG, and STG (Figure 7), which had been reported to associate with happy emotion in music.

According to the contrast analysis, the right STG and bilateral insula were included in the brain areas with stronger activation in the case of masked aircraft noise compared with aircraft noise (Table 2, Figure 4a,b). Similar results were presented in the previous fMRI studies on music. Sachs, Habibi, Damasio, and Kaplan (2018) found that the STG and anterior insula were overlapping brain activation areas under several kinds of music. They exhibited emotion‐specific and modality‐general patterns of neural activity and correlated with a behavior measure of empathy. Satoh et al. (2016) revealed that impaired fiber connectivity between the insula and STG might cause the musical anhedonia which reflected their important impact on emotion perception of music. On the other hand, in reality, the ability to discern music emotion is also common. Fruehholz's research supported that music without human voice could still express emotion (Fruehholz, Trost, & Grandjean, 2014). Even without any musical training, people could discern music emotion (happy, sad, and fearful) consistently and reliably (Fritz et al., 2009). Synthesizing these previous research, we might infer that subjects discerned the emotion in the masking music during the experiment and their empathy with the music was associated with the activation in STG and insula.

In addition, left medial frontal cortex and right medial OFC were also included in the brain areas with stronger activation in the case of masked aircraft noise compared with aircraft noise (Table 2, Figure 4a,b). The majority of the previous neuroscience studies indicated that the frontal lobe especially OFC were most directly associated with emotion (Hornak et al., 2003). Healthy people showed greater activation in OFC than depressed patients when listening to their favorite music which reflected OFC's association with positive emotion in music (Osuch et al., 2009). Different sides of the frontal lobe were linked with different kinds of emotion. Subjects' left frontal lobe was activated when listening to the delightful light music (Xiang, Zhang, & Zhang, 2006). Moreover, our results showed that the activation level in the left medial frontal cortex and right medial OFC had a significant negative correlation with subjective annoyance scoring (Figure 4c,d). These results suggest that the activation in the two frontal cortex are associated with positive emotion in the masking music, and we propose that one possible mechanism of using masking sound to decrease the noise annoyance is that music may pass positive emotion to people and thus help restrain the annoyance. In the functional connectivity analysis, we selected the two frontal cortex as the seed regions. When subjects listened to the masked aircraft noise, OFC showed significant functional connectivity with IOG, PHG, and bilateral STG (Figure 7). However, when they listened to the aircraft noise, the functional connectivity was unsignificant. In a research on neural processing of musical emotions, prefrontal and occipital areas were associated with the cognitive processing of music and emotion recognition and regulation, and left PHG was associated with happy emotion in music (Bogert et al., 2016), which were similar with our results.

On the contrary, some brain areas including CN and ACC showed weaker activation in the case of masked aircraft noise compared with the aircraft noise (Table 3, Figure 5a,b). In the field of neurology, there were hardly any fMRI research on the brain activaiton of noise annoyance. However, some other negative emotions were widely studied and our acitvation results of noise annoyance were in line with those of the negative emotions in previous study. For example, CN is one of the regions involved in mood and anxiety disorders (Aupperle, Sullivan, Melrose, Paulus, & Stein, 2011; Price & Drevets, 2012). Amemori reported that applying a microstimulation to the CN of the monkeys was capable of inducing a sharp and prolonged state influencing pessimistic valuation (Amemori, Amemori, Gibson, & Graybiel, 2018). ACC also had a close relationship with the processing of negative emotion such as anxiety and fear (Etkin, Egner, & Kalisch, 2011). It had also been found to be activated in the process of various induced negative emotions including sadness and anxiety in normal healthy volunteers and psychopath (Bush, Luu, & Posner, 2000). In a neurobiological models of adult depression, ACC was thought to be pivotal to affective regulation and depression (Connolly et al., 2013). Moreover, activation level in bilateral ACC had a significant positive correlation with subjective annoyance scoring (Figure 5c,d). In general, we may infer that the activation in CN especially ACC are associated with noise annoyance and the sound masking may decrease the noise annoyance through restraining the activation in bilateral ACC. In the functional connectivity analysis, bilateral ACC were selected as the seed regions. The strength of ACC‐PFC functional connectivity decreased with the annoyance increased (from the masked aircraft noise to the aircraft noise; Figure 6c,d). The increase of activation in ACC and decrease of ACC‐PFC functional connectivity were also reported in previous studies on negative emotions other than noise annoyance. Negative emotions like anxiety and worry led to inefficient high‐order control, characterized by insufficient ACC‐PFC functional connectivity. The activation in ACC was explained to be the compensation for deficient ACC‐PFC connectivity (Barker et al., 2018; Basten, Stelzel, & Fiebach, 2011; Comte et al., 2015). In addition, the strength of ACC‐PFC functional connectivity had a significant negative correlation with the subjective annoyance scoring (Figure 6e,f). In general, our results suggested that noise annoyance were associated with deficient functional connectivity in ACC‐PFC but exceeded brain activation in ACC, which might be explained as compensation.

EEG has been used to investigate sound masking for many years. The aims of these sound masking research are to understand speech recognition (Brett A. Martin, Kurtzberg, & Stapells, 1999; B. A. Martin & Stapells, 2005). The application scenarios includes attenuating distraction from background speech (Jahncke, Bjorkeholm, Marsh, Odelius, & Sorqvist, 2016), protecting the privacy of speech (Niemczak & Vander Werff, 2019) and speech understanding in the presence of concurring sound (Getzmann & Wascher, 2017). Some studies reported pronounced late positive complex and N400 components of auditory event‐related potential over frontal areas in “cocktail‐party” situation (Davis & Jerger, 2014; Getzmann, Hanenberg, Lewald, Falkensteinand, & Wascher, 2015). In these studies, the activities in the frontal area were considered to associate with inhibition of unwanted auditory stimuli and increased allocation of attention to the target speech. These activities in the frontal area revealed by EEG may be related to the brain activation in frontal areas revealed by fMRI. The frontal activation may be associated with inhibition of aircraft noise and increased attention to the masking sound.

At last, some limitations of this study and the possible further research directions should be noted. First, previous studies have shown that age might be an important factor that may influence people's perception and brain response to sound masking. Annoyance from noise has been shown to follow an inverted U‐shaped pattern as a function of age, where the youngest and oldest subjects reported the lowest levels of annoyance, and people in their mid‐40s reported the highest levels of annoyance (Van Gerven, Vos, Van Boxtel, Janssen, & Miedema, 2009). For masking sound, the elderly reported greater calmness when hearing natural sounds (Hedblom, Knez, Sang, & Gunnarsson, 2017) and stronger happiness when hearing happy music (Vieillard & Gilet, 2013) than did younger and middle‐aged individuals. However, these studies are mainly based on subjective psychological tests, and no study about the age factor in sound masking effect by fMRI has been reported yet. Since all the subjects are young adults (mean age = 26 years, standard deviation of age = 8.08 years), the results in this study can only shed light on the masking effect in young adults. The age factor in masking effect and its underlying mechanism is to be explored in the future. Second, there are also various acoustic characteristics that may influence masking effects. Studies based on subjective annoyance evaluation showed that masking effects could be influenced by several factors, such as pleasantness, eventfulness, and familiarity (Axelsson, Nilsson, & Berglund, 2010), and the SPL difference between noise and masking sound (Jeon et al., 2010). However, no study about the effect of the interior factors on sound masking was reported based on neuroimaging techniques. The inherent influence of these factors and the corresponding underlying neural mechanisms are valuable to be investigated with specially designed fMRI experiment in the future.

## 5 CONCLUSION

Based on these results, we infer that the mechanisms of sound masking are associated with two aspects. First, sound masking may pass positive emotion to people and thus help restrain the annoyance. The brain response appears as increased activaiton in the right STG, bilateral insula, left medial frontal cortex, right medial OFC, and a neural network including OFC, IOG, PHG, and STG. Second, noise annoyance is associated with deficient functional connectivity in ACC‐PFC but exceeded brain activation in ACC, which may be explained as compensation. Sound masking is associated with strong connectivity in ACC‐PFC and activation decrease in ACC. These changes in ACC and PFC may help relieve the annoyance. Taken together, this study offered new insights on the underlying neural mechanisms of sound masking and established the possible method to understand the relationship between the physiological responses and psychological perceptions.

## Data Availability

The authors declare that some or all data of the study are available from the corresponding author by reasonable request.
